# Effectiveness of Japanese traditional medicine (Kamikihito and Saikokeishito) for treating long COVID: a prospective observational study

**DOI:** 10.3389/fmed.2025.1609812

**Published:** 2025-07-21

**Authors:** Rie Ono, Shin Takayama, Ryutaro Arita, Kota Ishizawa, Akiko Kikuchi, Michiaki Abe, Minoru Ohsawa, Natsumi Saito, Takeshi Kanno, Koh Onodera, Tadashi Ishii

**Affiliations:** ^1^Department of Kampo Medicine, Tohoku University Hospital, Sendai, Miyagi, Japan; ^2^Department of Education and Support for Regional Medicine, Tohoku University Hospital, Sendai, Miyagi, Japan; ^3^Department of Anesthesiology and Perioperative Medicine, Tohoku University Hospital, Sendai, Miyagi, Japan; ^4^Department of Kampo and Integrative Medicine, Tohoku University Graduate School of Medicine, Sendai, Miyagi, Japan

**Keywords:** long COVID, brain fog, traditional medicine, comprehensive QOL, Kampo, saikokeishito (SAKT), kamikihito (KKT)

## Abstract

**Background:**

Long COVID symptoms, especially brain fog, significantly impair patient quality of life (QOL); however, effective treatments remain elusive. Japanese traditional medicine (JTM, usually called Kampo medicine) is often used adjunctively to treat patients with diverse manifestations of long COVID.

**Objective:**

To evaluate the effectiveness of JTM in treating long COVID using a comprehensive QOL assessment.

**Methods:**

This single-center, prospective observational study evaluated QOL changes in patients with symptoms persisting beyond 28 days from the onset of COVID-19 (long COVID) who visited our general medicine outpatient department between October 2021 and August 2024. The treatment plan was determined by the attending physician based on the patient’s condition. The health-related QOL (HR-QOL) was comprehensively assessed using EuroQol-5 demensions-5levels (EQ-5D-5L) scores (ranging from −0.025 to 1.000, with higher values indicating better HR-QOL) at baseline and 3 months after the first visit. The formulations and factors associated with QOL changes were analyzed using multivariate logistic regression analyses.

**Results:**

We analyzed 112 patients. The most common symptoms were fatigue (83.9%). The median (interquartile range) HR-QOL of the entire cohort significantly increased from 0.711 (0.561–0.711) at baseline to 0.833 (0.671–0.890) at 3 months (*p* < 0.0001); the proportion of patients exceeding the national standard significantly increased from 7.1% to 20.4% (*p* = 0.0037). The brain fog patients group (50.0%, *N* = 56), the median (interquartile range) HR-QOL of the entire cohort significantly increased from 0.677 (0.551–0.770) at baseline to 0.750 (0.623–0.846) at 3 months (*p* < 0.005). However, the proportion of patients achieving the Japanese average HR-QOL did not show improvement significantly. A total of 101 patients (90.2%) were treated with JTM, and a combination of kamikihito and saikokeishito was administered to 20 patients. Multivariate regression analysis revealed that the combination usage was associated with greater improvements in the HR-QOL in all patients (odds ratio 5.4) and brain fog patients’ group (odds ratio 6.1).

**Conclusion:**

Long COVID treatment involving JTM improved the patients’ QOL at 3 months. The combination of kamikihito with saikokeishito may be a potential treatment option for long COVID. However, a randomized controlled trial is required to confirm its efficacy.

## 1 Introduction

The emergence of severe acute respiratory syndrome coronavirus 2 (SARS-CoV-2) caused COVID-19, a pandemic that has had a significant global impact. Although the World Health Organization (WHO) declared the end of the public health emergency of international concern in May 2023 (1), ongoing infections with variant strains have led to over 776 million cases and 7 million deaths globally (2).

The condition known as “long COVID,” in which the symptoms persist beyond the acute phase of infection, has been recognized since the early stages of the COVID-19 pandemic. The prevalence of long COVID varies based on the survey methodology and the time of assessment, with reported rates ranging from 7 to 88% (3–5). Given that long COVID can develop even in mild cases that do not require hospitalization during the acute phase, and considering the large number of individuals infected worldwide, it is estimated that at least 70 million people have experienced long COVID symptoms to date.

COVID-19 is a systemic disease that can affect multiple organ systems, including the respiratory, neurological, cardiovascular, and gastrointestinal systems (6). Long COVID presents with a wide array of symptoms involving diverse bodily functions. Common symptoms include fatigue, headache, cognitive impairment (“brain fog”), musculoskeletal pain, breathlessness, olfactory and taste dysfunction, and insomnia (7–9).

These symptoms frequently lead to limitations or disturbances in daily life activities and social functioning (10), resulting in a decline in the quality of life (QOL) over time (11, 12). Notably, long COVID has been reported to pose a higher risk in younger individuals, leading to significant social implications. Despite numerous investigations, treatment strategies have primarily focused on rehabilitation and cognitive behavioral therapy in the absence of organic pathology (13). While some studies have suggested the efficacy of pharmacological interventions such as antidepressants (14), a definitive treatment has yet to be established. Therefore, long COVID continues to pose a significant medical problem (15–17), and the search for novel therapeutic strategies remains a critical public health issue.

The occurrence of postinfectious sequelae is not limited to COVID-19 (18). Japanese traditional medicine (JTM, usually called Kampo medicine) has been reported to be effective in patients with postinfectious conditions. Moreover, JTM is commonly employed for the management of psychiatric symptoms (19) and age-related issues such as frailty (20) and cognitive decline (21), which are also prevalent in long COVID. Additionally, JTM is integrated into the healthcare system, is covered by national insurance, and can be prescribed by physicians with licenses for Western medicine, leading to a high utilization rate of 86% (22). As a result, JTM is commonly used to manage long COVID symptoms (23, 24).

We have previously demonstrated that JTM was used in approximately 80% of patients with symptoms 1 month post-COVID-19 and that the prevalence of symptoms at 6 months was relatively low (25). Our previous study before the Delta variant was reported, showed that respiratory symptoms were the most common symptom of long COVID. However, the Omicron variant has led to a notable increase in the number of patients presenting with fatigue and brain fog, and those with multiple symptoms. In this study, we aimed to assess the impact of complementary JTM on the comprehensive quality of life (QOL) of patients with various symptoms of long COVID. Given that patients with brain fog experience a notable clinical reduction in QOL and present treatment challenges, we conducted separate analysis of this subgroup.

## 2 Materials and methods

### 2.1 Ethics

The study was conducted in accordance with the Declaration of Helsinki and Tokyo guidelines for human studies and was approved by the ethical committee of Tohoku University, Miyagi, Japan (approval number: 2021-447).

### 2.2 Setting

In this study, the term “long COVID” was defined according to the National Institute for Health and Care Excellence (NICE) criteria (UK), which have defined the following three clinical stages of conditions after COVID-19: acute COVID-19 (symptoms resolving within 4 weeks of onset), ongoing symptomatic COVID-19 (symptoms persisting between 4 and 12 weeks), and post-COVID conditions (symptoms persisting for 12 weeks or longer) (26). The latter two stages are collectively termed “long COVID.” In this study, patients were classified according to these definitions.

In Japan, COVID-19 has spread since January 2020. Subsequently, the Alpha variant became predominant in March 2021, the Delta variant in July 2021, and the Omicron variant in December 2021 (27, 28).

The department of general medicine at Tohoku University Hospital initiated care for patients with persistent COVID-19 conditions in March 2021. At their first visit, the patients underwent a comprehensive assessment, including appropriate diagnostic tests based on their presenting symptoms. Specialist referrals were made, as required. The decision regarding the use of Western medicine, JTM, or a combination of both was made by the attending physician, considering the patient’s condition and preferences. Treatment allocation was not randomized, and potential confounding by indication may have influenced the treatment effects. When JTM was used for treatment, all prescriptions were Extract Granules for Ethical Use (Tsumura & Co). JTM for ethical use is approved by the Ministry of Health, Labour and Welfare, and their manufacturing method complies with Japanese Pharmacopoeia as regards their component and quantity. The term Ethical use here means that a medicine can only be handed out to patients with a doctor’s prescription and the National Health Insurance will cover the expense. The Japanese Pharmacopoeia (The Japanese Pharmacopoeia seventeenth edition English version, 2021) officially defines the origin and description of the listed crude drugs and JTM extracts and elaborates on their limited values and testing methods. Detailed information on each medication is available on STORK.^1^ The dosage was set to the standard adult dose as specified in the package insert, and it was sometimes reduced depending on patients’ age, condition, and concurrent use of other JTM formulations.

Considering that patients with long COVID often experience multiple symptoms, a comprehensive score is important for evaluating treatment effectiveness. Consequently, to assess the impact of our interventions, patients completed the EuroQol-5D-5L (EQ-5D-5L) (29) questionnaires at baseline and the 3-month follow-up. EQ-5D-5L, a widely used instrument for assessing health status, consists of five dimensions: mobility, self-care, usual activities, pain/discomfort, and anxiety/depression. Each dimension is rated on a 5-point scale (1 = no problems, 2 = slight problems, 3 = moderate problems, 4 = severe problems, and 5 = extreme problems). The patient responses are then converted into a health-related QOL (HR-QOL) value using a standardized value set (ranges between −0.025 and 1.000, with higher values indicating better HR-QOL) (30). The Japanese standard values were established through a large-scale survey targeting individuals aged 16–89 years (31). The EuroQol Visual Analogue Scale (EQ-VAS) assesses a patient’s self-rated health status on a scale ranging from 0 (worst imaginable health state) to 100 (best imaginable health state).

### 2.3 Study design

This prospective observational study included patients who visited our general medicine outpatient department between March 2021 and August 2024 with complaints of COVID-19 sequelae. Patients were excluded if they lacked a confirmed COVID-19 diagnosis, presented within 4 weeks of symptom onset, were under 18 years of age, had a follow-up period shorter than 3 months, or lacked EQ-5D-5L data. We also excluded three patients without medication. The reason for this exclusion was as follows: Two patients tended to improve conditions at their initial visit, resulting in the clinical decision that medication was unnecessary. The remaining patient was excluded due to a temporary worsening of a prior psychiatric condition, for which treatment at our facility was not required.

The primary outcome was the difference in HR-QOL (ranges between -0.025 and 1.000; higher values indicate better HR-QOL) assessed at baseline and 3 months. The secondary outcomes included: the proportion of patients who achieved the Japanese average HR-QOL at 3 months; changes in EQ-VAS and individual dimensions levels; and changes in the proportion of patients reporting no or slight problems (level ≤ 2).

Next, we defined “improvement” as ΔQOL (HR-QOL [3 months] – HR-QOL [baseline]) being greater than 0.065. This threshold is based on previously reported minimally important differences (MID) for EQ-5D-5L (32, 33). We then explored multivariate analysis to identify factors associated with improvement.

A separate analysis was performed on patients with brain fog. The definition of brain fog in this analysis was based on a questionnaire used by our department. Patients were classified as having brain fog if they provided three or more positive responses to questions regarding cognitive impairment or they presented evident cognitive decline symptoms that impeded their daily functioning.

### 2.4 Statistical analyses

Continuous variables were presented as median (interquartile range (IQR): 25th–75th percentile). Comparisons between the values at baseline and 3 months were made using the Wilcoxon signed-rank test, whereas the Mann-Whitney U test was used for between-group comparisons. Categorical variables were expressed as the number of patients (%) and their values were compared using either chi-square or Fisher’s exact test.

Multivariate logistic regression analysis was performed to identify factors associated with QOL improvement. A comprehensive univariate logistic regression analysis was also performed to identify the potential patient outcome predictors. These included factors previously reported to be associated with long COVID: female sex, older age, high body mass index (BMI), vaccination, smoking, multiple comorbidities, psychiatric disorders (34, 35), the duration from symptom onset, and duration of symptoms (potential relevance to symptom changes). We further considered including variables with a p-value less than 0.1, comparing the improvement and non-improvement groups. Selecting explanatory variables for multivariate logistic regression analysis was based on: factors associated with symptom persistence in previous literature, the JTM prescriptions treatment effects, comprehensive univariate logistic regression analysis results, and each group sample size.

Statistical analyses were performed using Graph Pad Prism 10 for MacOS (version 10.4.0) (GraphPad Software, La Jolla, California, United States).

## 3 Results

**FIGURE 1:**
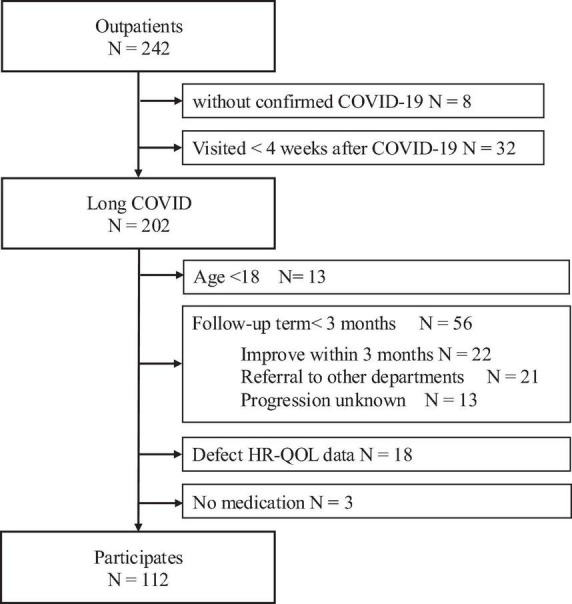
Flowchart describing patient enrollment. Of the 242 patients who visited our department during the study period, 112 were eligible for the analysis. The following patients were excluded: 8 without a confirmed diagnosis, 32 who presented with the symptoms <4 weeks after symptom onset, 13 under the age of 18 years, 56 with a follow-up period of <3 months (22 discharged following improvement, 21 transferred to other departments or hospitals, and 13 with unknown clinical course), 18 with missing QOL data, and 3 with no medication. HR-QOL, health related-quality of life.

Of the 242 patients who visited our department during the study period, 112 were eligible for the analysis. Figure 1 provides a detailed overview of patient enrollment.

Table 1 shows the baseline characteristics of all included patients, presented alongside those of patients with brain fog (*N* = 56). The patient background was characterized by a high proportion of working-age individuals, a predominance of non-hospitalized patients, and a majority of patients from the Omicron variant period.

**TABLE 1:** Clinical variables of all study participants.

Variable	*N* = 112
Characteristics	
Age: years median (IQR)	39 (26–49)
Sex, male, female: N (Female%)	54, 58 (51.8)
Body mass index (BMI): median kg/m^2^ (IQR)	22.9 (20.0–26.7) (NA 1)
Smoking (current): N (%)	33 (29.5) (NA 7)
Alcohol (habitual): N (%)	58 (51.8) (NA 11)
Vaccination: times (IQR)	3 (2–4) (NA 17)
Non-hospitalization in the acute phase: N (%)	110 (98.2)
Alpha variant dominant at acute phase: N (%)	3 (2.7)
Delta variant dominant at the acute phase: N (%)	6 (5.4)
Omicron variant dominant at acute phase: N (%)	104 (92.9)
Duration from the onset of COVID-19 to first visit outpatients: days: median (IQR)	102 (51–174)
**Comorbidities: N (%)**	
Digestive	34 (30.4)
Cardiovascular	25 (22.3)
Respiratory	25 (22.3)
Psychological	24 (21.4)
Neurology	19 (17.0)
Orthopedic	19 (17.0)
Genital	19 (17.0)
Dyslipidemia	14 (12.5)
Renal/urological	12 (10.7)
Immunology/Allergy	9 (8.0)
Diabetes mellitus	8 (7.1)
Metabolism/Endocrine	8 (7.1)
Malignant tumor	3 (2.7)
Others	30 (26.8)
Number of Comorbidities/one patient →: median (IQR)	2 (1–3)
**Laboratory data: median (IQR)**	
White blood cells: × 10^3^/μL	5.9 (5.0–6.9)
Hemoglobin: g/dL	14.3 (13.0–15.4)
Hematocrit:%	42.8 (40.5–46.2)
Platelet: × 10^3^μL	262 (231–299)
Total bilirubin: mg/dL	0.7 (0.6–0.9)
γ-glutamyltransferase: U/L	22 (15–40)
Aspartate aminotransferase: U/L	19 (17–26)
Alanine aminotransferase: U/L	21 (13–36)
Alkaline phosphatase: U/L	67 (58–80)
Lactate dehydrogenase: U/L	161 (147–180)
Blood Urea Nitrogen: mg/dL	12 (10–15)
Creatinine: mg/dL	0.72 (0.62–0.85)
Estimated glomerular filtration rate: mL/min/1.73 m^2^	83 (73–96)
Sodium: mEq/L	140 (139–141)
Potassium: mEq/L	4.2 (4.0–4.4)
Chlorine: mEq/L	104 (103–104)
Zinc: μg/dL	82.1 (74.3–88.8)
C-reactive protein: mg/dL	0.05 (0.01–0.12)
Ferritin: ng/mL	85.3 (34.9–189.2)
Erythrocyte sedimentation rate: mm	13 (5–21)
Cortisol: μg/dL	9.4 (6.7–11.5)
Adrenocorticotropic hormone: pg/mL	20.4 (14.5–30.0)

N, number; IQR, Interquartile range; NA, not applicable.

Table 2 shows the symptoms (visual analog scale ≥ 20) of long COVID at the initial visit. The median number of symptoms per patient was four. The most common symptom was fatigue, which was observed in 94 patients (83.9%). Brain fog was the third most common symptom.

**TABLE 2:** Long COVID symptoms of all patients.

Symptoms: N (%)	*N* = 112
Fatigue	94 (83.9)
Breathlessness	75 (54.5)
Brain fog	56 (50.0)
Headache	50 (44.6)
Musculoskeletal pain	39 (34.8)
Cough	26 (23.2)
Sputum	23 (20.5)
Taste dysfunction	25 (22.3)
Olfactory dysfunction	18 (16.1)
Abdominal discomfort	6 (4.5)
Psychological	6 (5.4)
Hair loss	6 (5.4)
Chest discomfort	4 (3.6)
Dizziness	2 (1.8)
Others	5 (3.6)

N, number; IQR, Interquartile range.

Formulation of JTM was used for 101 cases (90.2%), either alone or in combination with Western medicine. Among the 57 kinds used, the most frequently prescribed JTM formulations was saikokeishito (SAKT), followed by kamikihito (KKT) (Table 3 presents the 10 most frequently administered prescriptions). Multiple prescriptions of JTM were often used (median of 2 per patient), with the most prevalent combination being KKT and SAKT. The current prescription of SAKT was SAKT Extract Granules for Ethical Use (Tsumura & Co), wherein 7.5g of SAKT extract granules contain a dried extract of 9 crude drugs: Japanese Pharmacopeia (JP) Bupleurum Root (5.0g), JP Pinellia Tuber (4.0g), JP Scutellaria Root (2.0g), JP Glycyrrhiza (2.0g), JP Cinnamon Bark (2.0g), JP Peony Root (2.0g), JP Jujube (2.0g), JP Ginseng (2.0g), JP Ginger (1.0g) (The Japanese Pharmacopeia 18th edition, 2021) (Table 4a). The current prescription of KKT was KKT Extract Granules for Ethical Use (Tsumura & Co) (Table 4b). Saikokeishito (TJ-10) is indicated for the relief of the following symptoms of those patients with fever, diaphoresis, rigors, physical pain, headache, and nausea: febrile diseases, such as common cold, influenza, pneumonia, and pulmonary tuberculosis; and stomach pit tension pain, such as gastric ulcer, duodenal ulcer, cholecystitis, cholelithiasis, hepatic dysfunction, and pancreatitis. The current prescription of KKT was KKT Extract Granules for Ethical Use (Tsumura & Co) wherein 7.5g of KKT extract granules contain a dried extract of 9 crude drugs: JP Astragalus Root (3.0g), JP Bupleurum Root (3.0g), JP Jujube Seed (3.0g), JP Atractylodes Lancea Rhizome (3.0g), JP Ginseng (3.0g), JP Poria Sclerotium (3.0g), JP Longan Aril (3.0g), JP Polygala Root (2.0g), JP Gardenia Fruit (2.0g), JP Jujube (2.0g), JP Japanese Angelica (2.0g) Root, JP Glycyrrhiza (1.0g), JP Ginger (1.0g), JP Saussurea Root (1.0g). Kamikihito (TJ-137) is indicated for the relief of the following symptoms of those patients with delicate constitution and a poor complexion: anemia, insomnia, mental anxiety, and neurosis.

**TABLE 3:** Content of treatment for the symptoms.

Medications: N (%)	*N* = 112
Western monotherapy	11 (9.8)
Therapy using JTM	101 (90.2)
**Formulation details of JTM utilized: N (%)**	***N* = 101**
Saikokeishito	48 (42.9)
Kamikihito	29 (25.9)
Hochuekkito	12 (10.7)
Ninjin’yoeito	10 (8.9)
Goreisan	8 (7.1)
Saireito	7 (6.3)
Saibokuto	6 (5.4)
Ryokeijutsukanto	6 (5.4)
Keishibukuryogankayokuinin	6 (5.4)
Tokisyakuyakusan	5 (3.5)
**Combination utilized**	
Kamikihito, saikokeishito: N (%)	20 (14.9)
Ninjinyo’eto, saireito: N (%)	3 (3.0)
Kamikihito, saireito: N (%)	2 (1.8)

N, number; IQR, Interquartile range; JTM, Japanese traditional medicine.

**TABLE 4:** Outcome data: the change in parameters related EQ-5D-5L.

(a) The changes in HR-QOL and EQ VAS of all patients				
	First visit	3 months later	**Δ**	p
HR-QOL, median (IQR)	0.711 (0.561–0.711)	0.833 (0.671–0.890)	0.087 (0–0.18)	< 0.0001
Patients who achieved the Japanese average HR-QOL, N (%)	8 (7.1)	23 (20.4)		0.004
EQ VAS, median (IQR)	50 (36–64)	60 (45–80)	10 (-5–20)	< 0.0001
**(b) The changes in HR-QOL and EQ VAS of brain fog patients**				
	First visit	3 months later	**Δ**	p
HR-QOL, median (IQR)	0.677 (0.551–0.770)	0.750 (0.623–0.846)	0.075 (−0.055 to 0.158)	0.005
Patients who achieved the Japanese average HR-QOL, N (%)	1 (1.8)	6 (10.7)		0.11
EQ VAS, median (IQR)	45 (30–59)	53 (40–70)	7.5 (−9.3 to –24.3)	0.007

### 3.1 Improvements in the HR-QOL

**FIGURE 2:**
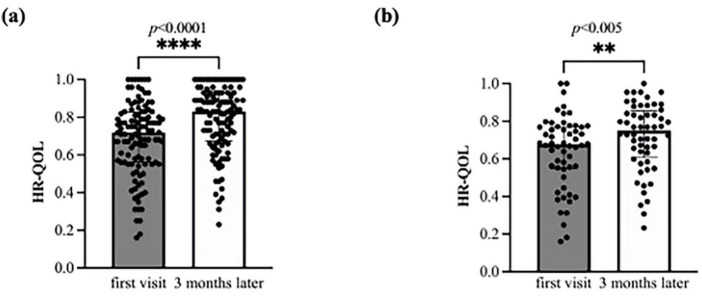
Bar charts of the health-related quality of life (HR-QOL) status at baseline and at 3 months. The HR-QOL ranged between −0.025 and 1.000; higher values indicate better HR-QOL. **(a)** All 112 study participants were evaluated for the change in HR-QOL from baseline to 3 months. The HR-QOL significantly improved from 0.711 at baseline to 0.833 at 3 months (*p* < 0.0001). **(b)** A total of 56 patients with brain fog were evaluated for the change in HR-QOL from baseline to 3 months. The HR-QOL significantly improved from 0.677 at baseline to 0.750 at 3 months (*p* = 0.005).

The initial HR-QOL was 0.711 (0.562–0.763), with eight patients (7.1%) above the national standard. After 3 months, the HR-QOL significantly improved to 0.833 (0.671–0.890) (*p* < 0.0001), a difference of 0.087 (0–0.177) surpassing the MID. The number of patients exceeding the national standard also significantly increased to 23 (20.4%) (*p* = 0.004) (Figure 2a, Table 4a). In addition, there was a change of 10 (−5–20) in the EQ-VAS, demonstrating statistically significant improvement (*p* < 0.0001).

Regarding each item on the questionnaire of EQ-5D-5L, the usual activity level demonstrated a significant reduction (*p* < 0.0001), and the number of patients with a level of 2 or less significantly increased from 45 to 69 (40.2% vs. 61.6%; *p* = 0.001) (Table 5a). Furthermore, pain/discomfort and anxiety/depression also exhibited a significant decrease in level (*p* < 0.0001, *p* < 0.0001) and a significant increase in the number of patients with a level ≤ 2 (46.4% vs. 67.0%; *p* = 0.002, 63.4% vs. 80.4%; *p* = 0.005).

**TABLE 5:** Outcomes for individual items of the EQ-5D-5L questionnaire.

(a) All patients			
Individual questionnaire	First visit	3 months later	p
**Mobility**			
Level, median (IQR)	2 (1–3)	1 (1–2)	0.002
Level ≤ 2, N (%)	77 (68.8)	85 (75.9)	0.23
**Self-care**			
Level, median (IQR)	1 (1–2)	1 (1–1)	0.09
Level ≤ 2, N (%)	103 (92.0)	103 (92.0)	> 0.99
**Usual activities**			
Level, median (IQR)	3 (2–4)	2 (1–3)	< 0.0001
Level ≤ 2, N (%)	45 (40.2)	69 (61.6)	0.001
**Pain/discomfort**			
Level, median (IQR)	3 (2–3)	2 (1–3)	< 0.0001
Level ≤ 2, N (%)	52(46.4)	75 (67.0)	0.002
**Anxiety/depression**			
Level, median (IQR)	2 (2–3)	2 (1–2)	< 0.0001
Level ≤ 2, N (%)	71 (63.4)	90 (80.4)	0.005
Shows the median level and the number of responses with a level of 2 or less for each individual questionnaire (1 = no problems, 2 = slight problems, 3 = moderate problems, 4 = severe problems, and 5 = extreme problems)			
**(b) sBrain→Brain fog patients**			
**Mobility**			
Level, median (IQR)	2 (1–3)	2 (1–3)	0.097
Level ≤ 2, N (%)	36 (64.3)	38 (67.9)	0.69
**Self-care**			
Level, median (IQR)	1 (1–2)	1 (1–1)	0.48
Level ≤ 2, N (%)	51 (91.1)	49 (87.5)	0.54
**Usual activities**			
Level, median (IQR)	3 (2–4)	3 (2–3)	0.0004
Level ≤ 2, N (%)	17 (30.4)	27 (48.2)	0.053
**Pain/discomfort**			
Level, median (IQR)	3 (2–4)	2 (2–3)	0.0003
Level ≤ 2, N (%)	21 (37.5)	33 (58.9)	0.02
**Anxiety/depression**			
Level, median (IQR)	3 (2–4)	2 (2–3)	0.0006
Level ≤ 2, N (%)	26 (46.4)	30 (53.6)	0.007
Shows the median level and the number of responses with a level of 2 or less for each individual questionnaire (1 = no problems, 2 = slight problems, 3 = moderate problems, 4 = severe problems, and 5 = extreme problems)			

EQ-5D-5L, EuroQol 5 dimensions 5 levels; HR-QOL, health-related quality of life; N, number; IQR, Interquartile range; EQ VAS, EuroQol Visual Analogue Scale; Δ = data (3 months) – data (baseline).

In the subgroup analyses, the brain fog group showed a significant improvement in the HR-QOL (*p* = 0.005) and the EQ VAS (*p* = 0.007) (Figure 2b; Table 4b). Although the proportion of patients with HR-QOL above the national standard did not increase significantly (*p* = 0.11), regarding each item on the EQ-5D-5L questionnaire, the usual activities dimension demonstrated a significant reduction (*p* = 0.0004). However, the proportion of patients with a level of 2 or less did not change significantly (*p* = 0.053) (Table 5b). Conversely, the pain/discomfort and anxiety/depression dimensions exhibited a significant decrease in level (*p* = 0.0003 and 0.0006, respectively) and a significant increase in the proportion of patients with a level ≤ 2 (*p* = 0.02 and 0.007, respectively).

### 3.2 KKT/SAKT combined usage as candidates for long COVID treatment

The improved group (ΔQOL > 0.065) comprised 64 individuals (Supplementary table 1). The results of the univariate analysis showed that KKT/SAKT combined usage was a factor associated with QOL improvement (*p* = 0.03) (Table 6a). Multivariate analysis was conducted using the following factors: age, vaccination, duration from the onset of COVID-19 to the first outpatient visit, and KKT/SAKT combination usage.

**TABLE 6a:** Comprehensive univariate logistic regression analysis to identify potential predictors of the improvement of HR-QOL.

Variable	Odds ratio	95%CI	p
**Characteristics**			
Advanced age	1.02	0.99–1.05	0.24
Female gender	0.98	0.46–2.1	0.96
High body mass index	0.97	0.89–1.1	0.50
Current smoking	0.72	0.20–5.1	0.56
Frequent vaccination	1.2	0.92–1.6	0.19
Extended duration from the onset of COVID-19 to first visit outpatients	0.999	0.997–1.001	0.36
**Comorbidities**			
Psychological disease	1.2	0.46–2.9	0.74
Multiple comorbidities	0.94	0.76–1.2	0.58
**Laboratory data**			
High ferritin level	1.002	0.9991–1.006	0.20
**Symptoms**			
Headache	1.9	0.91–4.2	0.09
**Medications**			
Saikokeishito	2.0	0.93–4.4	0.08
Kamikihito/saikokeishito combined	3.7	1.2–13.6	**0.03**

**TABLE 6b:** Comprehensive univariate logistic regression analysis to identify potential predictors of the improvement of HR-QOL (brain fog patients).

Variable	Odds ratio	95%CI	p
**Characteristics**			
Advanced age	1.04	1.001–1.080	0.051
Female gender	0.64	0.22–1.8	0.41
High body mass index	1.15	1.007–1.3	0.05
Current smoking	1.7	0.38–9.3	0.41
Frequent vaccination	1.5	0.96–2.4	0.09
Extended duration from the onset of COVID-19 to first visit outpatients	1.00	0.998–1.003	0.78
**Comorbidities**			
Cardiovascular disease	5.1	1.2–36.3	0.0504
Psychological disease	0.83	0.23–2.8	0.76
Neurological disease	4.4	0.97–31.0	0.08
Multiple comorbidities	1.3	0.95–1.8	0.12
**Laboratory data**			
High ferritin level	1.01	1.005–1.02	0.008
**Medications**			
Saikokeishito	3.2	1.08–9.9	0.04
Kamikihito	2.6	0.87–8.0	0.09
Kamikihito/saikokeishito combined	4.8	1.4–19.5	0.02

HR-QOL, health related quality of life; CI, confidence interval.

**FIGURE 3:**
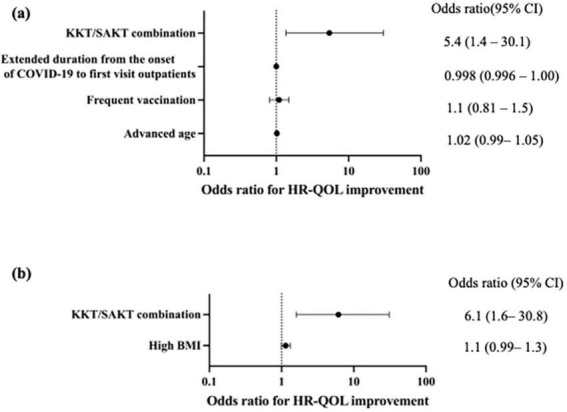
Forest plots of associations between medications and health-related quality of life (HR-QOL) outcomes. **(a)** Kamikihito (KKT) and saikokeishito (SAKT) combination use within 3 months was significantly associated with HR-QOL improvement (OR: 5.4, 95% CI: 1.4–30.1, *p* = 0.029). **(b)** Kamikihito (KKT) and saikokeishito (SAKT) combination use within 3 months were significantly associated with HR-QOL improvement (OR: 6.1, 95% CI: 1.6–30.8, *p* = 0.014). HR-QOL, health related-quality of life; CI, confidence interval; KKT, kamikihito; SAKT, saikokeishito; BMI, body mass index.

The odds ratio (OR) for QOL improvement with KKT/SAKT combination usage within 3 months of treatment was 5.4 (95% confidence interval [CI]: 1.4–30.1, *p* = 0.03) (Figure 3a)

In the subgroup analyses, the brain fog group had 30 individuals in the improvement group (Supplementary table 2). Univariate analysis suggested a potential association between the KKT/SAKT combination usage (*p* = 0.02) and high ferritin levels (*p* = 0.008) with QOL (Table 6b). However, considering previous reports, we conducted a multivariate analysis with KKT/SAKT combination usage and high BMI as variables. As a result, the OR for QOL improvement with KKT/SAKT combination usage within 3 months of treatment was 6.1 (95% CI: 1.6–30.8) (*p* = 0.01) (Figure 3b).

## 4 Discussion

In Japan, the integrated practice of traditional and Western medicine allows for healthcare that combines the strengths of both approaches. Given the diverse pathophysiological conditions that characterize long COVID, and the symptoms resulting from these conditions, an approach leveraging the advantages of both medical systems may be the most effective for patient treatment.

This study assessed the HR-QOL changes in patients with long COVID with a high prevalence of fatigue and found a significant improvement in the HR-QOL at 3 months, and the observed difference was greater than the MID. We noted a significant increase in the proportion of patients exceeding the national standard value (7.1% vs. 20.4%). JTM was used in 90.2% of patients, and treatments that included KKT/SAKT combination related to an improvement in HR-QOL (OR 5.4).

For patients with BF, the initial HR-QOL was observed to be low, and there was no statistically significant increase in the proportion of patients achieving the national standard. Nevertheless, a statistically significant improvement in HR-QOL was noted. The etiology of brain fog remains unclear and effective treatments are lacking, despite ongoing research. We observed symptomatic improvement in these patients at the 3-month follow-up. KKT/SAKT combination usage is related to the improvement of HR-QOL (OR 6.1). A conservative estimate of the prevalence of brain fog as 10% (reported range 10–40%) (36, 37) in patients with long COVID translates to a number needed-to-treat of 3.3. Thus, administration of KKT/SAKT combination to approximately three patients could be expected to result in improvement in one patient. This suggests a potential benefit, given the current lack of established treatments for brain fog. We report here, for the first time, the potential efficacy of KKT/SAKT combination usage for brain fog in this patient population. As treatment options for long COVID are still being explored, this finding warrants further comparative research in future studies.

Long COVID is characterized by a wide range of symptoms and several potential underlying mechanisms have been proposed, including persistent viral infection (38, 39), mitochondrial dysfunction (40, 41), immune dysregulation (42, 43), endothelial dysfunction (44), neuroinflammation (45, 46), and gut microbiota dysbiosis (47). Neuroinflammation-induced astrocyte alternation contributes to blood-brain barrier dysfunction (48), neuroendocrine system dysregulation (49), and disturbances in brain metabolism (50), leading to the development of neuropsychiatric symptoms. Furthermore, SARS-CoV-2 infection results in cognitive complaints and neuropsychological alterations through structural and functional alterations in the hippocampus and related cortices (51).

KKT is a formulation that is indicated for insomnia and mental anxiety in patients with a state of energy deficiency that impairs bodily functions (“Qi deficiency”; a traditional East Asian medical concept) (52). KKT and its constituent crude drugs may have potential efficacy against the multiple mechanisms underlying long COVID. KKT has been reported to mitigate stress responses through the activation of oxytocin neurons and receptors that orchestrate social and emotional behaviors (53). KKT promotes hippocampal neurogenesis, thereby exhibiting antidepressant and cognitive-enhancing effects (54). Additionally, Bupleuri Radix, a constituent crude drug of KKT, has demonstrated neuroprotective and anti-inflammatory effects (55). Ginseng Radix has been studied for its immunomodulatory properties and its influence on the gut microbiome and gut-associated immune system (56). Glycyrrhiza has been reported to exhibit anti-SARS-CoV-2 activity (57).

SAKT is prescribed to patients with symptoms of subacute postinfectious cold-like conditions, such as mild fever, headache, musculoskeletal pain, and gastrointestinal symptoms. Bupleuri Radix, Ginseng Radix, and Glycyrrhiza are also constituent crude drugs of SAKT. Furthermore, Baicalin, a component of Scutellariae Radix, which is a constituent crude drug of SAKT, has been shown to exhibit anti-SARS-CoV-2 activity (58) and suppress microglial activation *in vitro* (59).

KKT or SAKT monotherapy often demonstrates clinical effectiveness. A prior case report has elucidated the effectiveness of JTM treatment, including KKT, for fatigue accompanied by decreased concentrations, which suggests the positive effects of KKT (60). In a case series, we reported that SAKT was effective against fatigue without brain fog following COVID-19 infection (61). However, the treatment showed limited effectiveness in patients with severe brain fog symptoms. Combining these formulations led to noticeable symptom relief, as a result, the number of prescriptions gradually escalated. This suggests that SAKT and KKT functioned additively, enhancing their effects by sharing some crude drugs without any antagonistic interactions.

While JTM usually emphasizes individual constitutions, the results of this study provided preliminary insights into the potential general efficacy of the combination of KKT and SAKT in the management of long COVID.

On the other hand, in this study, a wide range of JTM formulations apart from KKT and SAKT were utilized due to the multifarious concurrent symptoms. It has been reported that long COVID symptoms can be categorized into distinct patterns (62). Future investigation may identify tailored prescriptions corresponding to each of the patterns.

However, we emphasize that long COVID should not be managed solely with JTM. Indeed, combination medication constituted 65.2% of the therapeutic interventions. COVID-19 has been reported to be associated with the onset and exacerbation of diseases such as diabetes and autoimmune disorders (63, 64). We observed frequent occurrences of exacerbation of comorbidities and development of new organic diseases. Western medicine is superior for the differentiation and treatment of these conditions, whereas JTM often provides relief for symptoms unresponsive to Western medicine or symptoms with unknown causes. Therefore, a combination of Western and traditional medical approaches is thought to be the optimal strategy for managing patients with diverse long COVID symptoms.

When using JTM formulations, especially in instances of multiple drug administration, it is necessary to pay attention to potential adverse effects, just as with those of Western medications. Glycyrrhizin, a major constituent of JTM formulations, carries the risk of pseudoaldosteronism, where high dosage, long-term use, and older age are constitutional risk factors (65). Daily dosage should be limited, and its use is contraindicated in patients with hypokalemia or uncontrolled hypertension. Among JTM, formulations containing Scutellariae Radix, a component of SAKT have a relatively high frequency of adverse effects (66), such as liver dysfunction or interstitial pneumonia (67). Regular examinations are conducted to monitor for the adverse effects during JTM administration. If the symptoms are relieved, a gradual dose reduction and discontinuation of administration should be considered.

This study has several limitations. The possibility of natural recovery in patients cannot be excluded, given that this study did not use a control group, and MID can vary depending on the disease. Owing to the limited number of cases in the BF group, the number of factors that could be included in the multivariate analysis was restricted. Furthermore, effective utilization of traditional medicine necessitates specialized knowledge, and variations in physician proficiency may have influenced the observed effects. Additionally, the study did not isolate the effects of KKT/SAKT combined usage from those of combination therapy with Western medicine. However, we believe that the add-on effect of KKT/SAKT can still be evaluated. These limitations warrant future randomized controlled trials.

## 5 Conclusions

Considering the diverse pathophysiological conditions and symptoms of long COVID, we believe that an approach that leverages the advantages of both Western medicine and JTM may be a potentially effective strategy. Furthermore, effective therapies for long COVID and brain fog have not yet been established. Our finding, that the use of a combination of KKT and SAKT improved the comprehensive QOL, is of considerable importance as it points towards the possibility of new treatment options. However, a well-designed randomized controlled trial with a larger sample size and longer follow-up is needed to validate its efficacy and determine the appropriate dosing and indications.

## Data Availability

The raw data supporting the conclusions of this article will be made available by the authors, without undue reservation.

## Footnotes

1
http://mpdb.nibiohn.go.jp/stork/
