# Supplementary material for: Effectiveness of Japanese traditional medicine (Kamikihito and Saikokeishito) for treating long COVID: a prospective observational study
Source: Front Med (Lausanne). 2025 Jul 21;12:1609812. doi: 10.3389/fmed.2025.1609812 (PMC12320050; doi:10.3389/fmed.2025.1609812)
Supplement: Supplementary file 1 [file Table_1.docx]

| **Supplementary Table 1: Comparisons of improvement and non-improvement groups (All patients)** | | | | | | |
| --- | --- | --- | --- | --- | --- | --- |
|  | |  |  |  |  |  |
| **Variable** | | **Non-improvement (N=48)** | | **Improvement (N=64)** | | **p** |
| Age: years median (IQR) | | 39 | (25–48) | 40 | (30–51) | 0.28 |
| Sex, male, female: N (Female%) | | 25, 23 | (52.1) | 33, 31 | (51.6) | 0.96 |
| Body mass index (BMI): median kg/m^2^ (IQR) | | 23 (NA 1) | (219.8–27.3) | 22.8 | (20.0–26.5) | 0.64 |
| Smoking (current): N (%) | | 7 (NA 3) | (14.6) | 7 (NA 4) | (10.9) | 0.56 |
| Alcohol (habitual): N (%) | | 14 (NA 5) | (29.2) | 19 (NA 6) | (29.7) | 0.95 |
| Vaccination: times (IQR) | | 3 (NA 8) | (1–3) | 3 (NA 9) | (2–4) | 0.12 |
| Hospitalization in the acute phase: N (%) | | 1 | (2.1) | 1 | (1.6) | >0.99 |
| Duration from onset of COVID-19 to first visit outpatients, days: median (IQR) | | 109 | (58–213) | 94 | (44–132) | 0.14 |
| Alpha variant dominant at acute phase; N (%) | | 3 | (6.2) | 0 | (0.0) |  |
| Delta variant dominant at acute phase | | 2 | (4.2) | 4 | (6.2) | 0.15 |
| Omicron variant dominant at acute phase | | 43 | (89.6) | 60 | (93.8) |  |
| Comorbidities: N (%) | |  |  |  |  |  |
| Digestive | | 17 | (35.4) | 17 | (26.6) | 0.31 |
| Respiratory | | 10 | (20.8) | 15 | (23.4) | 0.74 |
| Cardiovascular | | 9 | (18.8) | 16 | (25.0) | 0.43 |
| Psychological | | 11 | (22.9) | 13 | (20.3) | 0.74 |
| Neurology | | 7 | (14.6) | 12 | (18.8) | 0.56 |
| Genital | | 10 | (20.8) | 9 | (14.1) | 0.34 |
| Orthopedic | | 7 | (14.6) | 12 | (18.8) | 0.56 |
| Dyslipidemia | | 7 | (14.6) | 7 | (10.9) | 0.56 |
| Renal/urological | | 6 | (12.5) | 6 | (9.4) | 0.60 |
| Number of Comorbidities : /one patient, median (IQR) | | 2 | (1-4) | 2 | (1-3) | 0.80 |
| Laboratory data: median (IQR) | | | | | | |
| Ferritin: ng/mL | 55.1 (NA 5) | | (29.6–135.3) | 132.4 (NA 4) | (35.4–218.0) | 0.04 |
| Zinc: µg/dL | 79.7 (NA 8) | | (73.1–89.1) | 83.0 (NA 19) | (74.3–88.6) | 0.57 |
| Cortisol: µg/dL | 9.5 (NA 5) | | (6.4–12.9) | 9.3 (NA 5) | (6.8–11.0) | 0.58 |
| Erythrocyte sedimentation rate (ESR): mm | 12 (NA 3) | | (5–19) | 12 NA 4) | (5–24) | 0.53 |
| Symptoms, N (%) |  | |  |  |  |  |
| Fatigue | 38 | | (79.2) | 56 | (87.5) | 0.23 |
| Breathlessness | 22 | | (45.8) | 39 | (60.9) | 0.11 |
| Brain fog | 26 | | (54.2) | 30 | (46.8) | 0.45 |
| Headache | 17 | | (35.4) | 33 | (51.6) | 0.09 |
| Musculoskeletal pain | 17 | | (35.4) | 22 | (34.4) | 0.91 |
| Cough | 10 | | (20.8) | 16 | (25.0) | 0.61 |
| Sputum | 10 | | (20.8) | 13 | (20.3) | 0.95 |
| Taste dysfunction | 8 | | (16.7) | 17 | (26.6) | 0.21 |
| Olfactory dysfunction | 8 | | (16.7) | 10 | (15.6) | 0.88 |
| Number of symptoms, N/one patient, median (IQR) | 3 | | (2–5） | 4 | (3–5) | 0.14 |
| Medication; N (%) |  | |  |  |  |  |
| Therapy using JTM | 42 | | (87.5) | 59 | (92.2) | 0.41 |
| Saikokeishito | 16 | | (33.3) | 32 | (50.0) | 0.08 |
| Kamikihito | 10 | | (20.8) | 19 | (29.7) | 0.29 |
| Hochuekkito | 6 | | (12.5) | 6 | (9.4) | 0.60 |
| Ninjin'yoeito | 5 | | (10.4) | 5 | (7.8) | 0.74 |
| Kamikihito/saikokeishito combined | 4 | | (8.3) | 16 | (25.0) | 0.03 |
| Improvement: HR-QOL (3 months) – HR-QOL (baseline) > 0.065  N; number, IQR; Interquartile range | | | | | | |
| HR-QOL: health related quality of life, EQ VAS: EuroQol Visual Analogue Scale | | | | | | |
| NA: not applicable | | | | | | |

| **Supplementary Table 2: Comparisons of improvement and non-improvement groups (Brain fog patients)** | | | | | |
| --- | --- | --- | --- | --- | --- |
| **Variable** | **Not improvement (N = 26)** | | **Improvement (N = 30)** | | **p** |
| Age, years median (IQR) | 32 | (25–47) | 44 | (33–58) | 0.04 |
| Sex, male, female, N (Female%) | 11, 15 | (57.7) | 16, 15 | (46.7) | 0.41 |
| Body mass index (BMI), median kg/m^2^ (IQR) | 20.9 (NA 1) | (19.3–25.3) | 24.2 | (21.0–28.4) | 0.04 |
| Smoking (current), N (%) | 3 | (11.5) | 5 (NA 3) | (16.7) | 0.71 |
| Alcohol (habitual), N (%) | 7 | (26.9) | 13 (NA 2) | (43.4) | 0.20 |
| Vaccination, times (IQR) | 2 (NA 6) | (1–3) | 3 (NA 5) | (3–4) | 0.049 |
| Hospitalization in the acute phase, N (%) | 1 | (3.9) | 0 | (0.0) | 0.46 |
| Duration from onset of COVID-19 to first visit outpatients, days, median (IQR) | 135 | (71–264) | 122 | (62–427) | 0.87 |
| Comorbidities: N (%) |  |  |  |  |  |
| Digestive | 9 | (34.6) | 11 | (36.7) | 0.87 |
| Respiratory | 4 | (15.4) | 9 | (30.0) | 0.22 |
| Cardiovascular | 2 | (7.7) | 9 | (30.0) | 0.047 |
| Psychological | 6 | (23.1)] | 8 | (26.7) | 0.76 |
| Neurology | 2 | (7.7) | 8 | (36.7) | 0.06 |
| Genital | 7 | (26.9) | 3 | (10.0) | 0.16 |
| Orthopedic | 6 | (23.1)] | 9 | (30.0) | 0.36 |
| Number of Comorbidities : /one patient, median (IQR) | 2 | (0-4) | 3 | (2-4) | 0.09 |
| Laboratory data: median (IQR) | | | | | |
| Ferritin: ng/mL | 53.8 (NA 4) | (27.2–107.0) | 142.8 (NA 5) | (61.3–216.5) | 0.01 |
| Zinc: µg/dL | 76.7 (NA 6) | (69.9–86.5 | 78.4 (NA 10) | (70.8–87.1) | 0.64 |
| Cortisol: µg/dL | 10.3 (NA 3) | (5.9–14.5) | 9.4 (NA 4) | (6.6–11.5) | 0.52 |
| Erythrocyte sedimentation rate (ESR): mm | 9 (NA 1) | (4–18) | 11 (NA 4) | (4–26) | 0.52 |
| Symptoms; N (%) |  |  |  |  |  |
| Fatigue | 24 | (92.3) | 30 | (100) | 0.12 |
| Breathlessness | 12 | (46.2) | 19 | (63.3) | 0.20 |
| Headache | 10 | (38.5) | 17 | (56.7) | 0.17 |
| Musculoskeletal pain | 9 | (34.6) | 14 | (45.7) | 0.36 |
| Cough | 8 | (30.8) | 5 | (16.7) | 0.21 |
| Sputum | 7 | (26.9) | 6 | (20.0) | 0.54 |
| Taste dysfunction | 3 | (11.5) | 7 | (23.3) | 0.31 |
| Number of symptoms, N/one patient, median (IQR) | 4 | (3–5) | 4 | (4–6) | 0.26 |
| Medication: N (%) |  |  |  |  |  |
| Therapy using JTM | 24 | (92.3) | 29 | (96.7) | 0.59 |
| Saikokeishito | 11 | (42.3) | 21 | (70.0) | 0.04 |
| Kamikihito | 8 | (30.8) | 16 | (53.3) | 0.09 |
| Kamikihito/saikokeishito combined | 4 | (15.4) | 14 | (46.7) | 0.02 |
| Improvement: HR-QOL (3 months) – HR-QOL (baseline) > 0.065 | | | | | |
| IQR; Interquartile range, N; number | | | | | |
| NA: not applicable | | | | | |
| JTM: Japanese traditional medicine | | | | | |
